# Do No Harm in refugee humanitarian aid: the case of the Rohingya humanitarian response

**DOI:** 10.1186/s41018-021-00093-9

**Published:** 2021-03-08

**Authors:** Abu Faisal Md. Khaled

**Affiliations:** Dept. of International Relations, Bangladesh University of professionals (BUP), Dhaka, Bangladesh

**Keywords:** Host community, Rohingya, Refugee, Humanitarian aid, Localisation

## Abstract

The article broadly examines how humanitarian aid for Rohingya refugees inadvertently harmed poorer hosts and adversely affected local capacities for peace. The article also discusses possible ways of easing tension and improving social cohesion in the refugee-hosting areas, while also highlighting how policy- and mandate-related constraints hinder a humanitarian response anchored in the "Do No Harm" principle. Finally, the article concludes with the argument that the humanitarian agencies should not just limit themselves to identifying the unintended consequences and lapses in the intervention. Instead, the Do No Harm principle should lead humanitarian aid agencies to make an active effort to accept responsibility for the harm while taking all necessary steps to mitigate or avoid harming in future interventions.

## Introduction

On 25 August 2017, when the Arakan Rohingya Salvation Army (ARSA) launched an assault on approximately thirty police outposts and a military camp in northern Rakhine State, the long-smouldering conflict between the Myanmar military and the Rohingya ethnic minorities of Myanmar’s Rakhine state escalated (Reuters 2019). This assault was followed by Myanmar army’s systematic killing, raping, looting, and villages’ rasing. The violent retaliation forced over 700,000 people, mostly Rohingya Muslims, to seek refuge in neighbouring countries, mainly Bangladesh (Human Rights Watch 2019). Almost all the refugees fleeing persecution sought refuge, particularly around Ukhiya and Teknaf sub-districts of Cox’s Bazar^1^. As a result of the influx, the Kutupalong Refugee camp became the largest refugee camp globally, with more than 600,000 refugees living in an area of just 13 km^2^ (UNHCR 2018).

The Rohingya crisis resulted from a conflict, but such a massive refugee influx can be the catalyst for a new dimension of conflict in areas hosting refugees. The tension between the refugees and adversely affected hosts may escalate into conflicts around refugee camps where different social, demographic, institutional, and economic factors weakened the ability to adapt a strategic path to a conflict-sensitive humanitarian response and peaceful co-existence. Therefore, humanitarian agencies responding to the Rohingya emergency, a relatively peaceful humanitarian crisis, refer to the humanitarian principles, since a massive refugee settlement is often an ideal situation for potential conflict (Bryant and Wake 2018; United Nations 2020).

Refugees are persons of concern to the United Nations High Commissioner for Refugees (UNHCR) and its implementing partners (UNHCR 2017). However, the arrival of aid for the refugees has mixed implications for the impoverished refugee-hosting communities (Kok 1989; Jacobsen 1994; Jacobsen 2006; Maystadt and Verwimp 2014; Taylor et al. 2016). Humanitarian agencies keep refugees at the centre of their attention (Chambers 1986; Jacobsen 1996). Addressing adverse impacts of refugee settlements and refugee aid on poorer hosts is generally not the priority of humanitarian agencies. Consequently, humanitarian aid in a refugee-hosting region risks overlooking poorer hosts and harming them on multiple fronts (Aukot 2003). Such a situation underlines the significance and potentially divisive role of refugee humanitarian aid. As Anderson (1999) argued, when “assistance is given in the context of a conflict, it becomes part of the context and thus also of the conflict” (p. 01). Similarly, humanitarian aid given in the context of the Rohingya crisis is part of the overall context.

Against this backdrop, by adopting the "Do No Harm" principle as an analytical framework, the article broadly examines how humanitarian aid for Rohingya refugees inadvertently harmed poorer hosts and adversely affected local capacities for peace. In the process, the author also discussed possible ways of easing tension and improving social cohesion, while also highlighting how policy- and mandate-related constraints hinder a humanitarian response anchored in the Do No Harm principle. The author argues that actualisation of the Do No Harm principle in refugee response is particularly important against the backdrop of a large-scale, protracted refugee crisis as dimensions of refugee aid’s impacts are interrelated, often resulting in unintended consequences.

This study is based on extensive fieldwork divided into multiple phases spanning from March 2019 to June 2020 in Tenkaf and Ukhiya sub-districts of Cox’s Bazar district. A qualitative data collection approach, including open-ended interviews and small group discussion sessions and the personal observation of author, has been used to gain insights into the contrasting perspectives of the relevant stakeholders and essence of the effects they are currently facing due to the transfer of refugee humanitarian aid. Besides, the author participated in various types of seminars and community meetings to understand the dynamics at play between different stakeholders. The interview and discussion results have been cross-checked with other related research documents, publications, and news reporting. A detailed analysis of the related literature and empirical observation of the lives and livelihoods of the host communities and refugees and several years of experience working in Cox’s Bazar as a researcher and practitioner were useful in carrying out this study.

## Socio-economic context of the refugee hosting areas

Refugee aid agencies are required to examine the socio-economic dynamics of the areas hosting refugees before the unveiling of humanitarian program, as factors in the context of a post-refugee settlement, such as the pattern of demography, distribution of resources, environmental landscape, and accessibility to services and work opportunities, largely determine the complex interactions between local communities, aid agencies, and refugees. Thus, humanitarian aid agencies need to delineate various characteristics of the project site in finer details. As Anderson (1999) argued, “every aid project site is local and special” (p. 02).

The district of Cox’s Bazar is below the national average in terms of various social indicators, with around 33% of the population living in abject poverty, while Teknaf and Ukhiya are among the 50 most socio-economically disadvantaged sub-districts of the country (ACAPS-NPM Analysis Hub 2018; ISCG 2018). The refugee-hosting area’s food security and nutritional status are less than national standards, with most residents depending on daily wages and insufficient social safety measures (ISCG 2018). Furthermore, the area is vulnerable to the impact of natural disasters and climate change. Agriculture is the mainstay of livelihoods in Cox’s Bazar. The reliance of Teknaf on agricultural production is a whopping 81%, whereas Ukhiya’s dependency is 63% (UNDP 2018). According to the same UNDP report, as many as 92% of households depend mainly on firewood for cooking in Cox’s Bazar. Even before the refugees’ arrival, the region had inadequate cultivable land, leading to low agricultural production, higher food spending, and economic uncertainty.

Since the onset of the refugee arrival, both locals and refugees have been competing in the same unskilled daily labour market, leading to a significant decline in work opportunities for the host population’s marginalised and ultra-poor members. Compared to the pre-influx phase, local hosts engaged in the unskilled labour sector are found to be poorer, while skilled wage earners and some traders benefited from the resource flow (The Daily Star, 2019a; IOM 2018). Consequently, between August 2017 and May 2018, the average income of daily wage earners decreased by around 24%, raising the rate of poverty among the local population significantly (The World Bank 2019, p. 386).

Although the Government of Bangladesh and aid agencies have made an effort to limit the humanitarian crisis’ adverse impact on the local inhabitants, evidence suggests that poverty, social tension, and conflicts between refugees and hosts have increased (ACAPS-NPM Analysis Hub 2018). Recently, despite the criticism from the United Nations (U.N.) and donor agencies, hundreds of Rohingya refugees have been relocated by the Government of Bangladesh (GoB) to Bhashan Char, a low-lying island vulnerable to floods and cyclones (Beech 2020). The deteriorated security environment in Teknaf and Ukhiya of Cox’s Bazar district and “intense public pressure to resolve the Rohingya crisis” have been described as among the major factors behind this relocation (Abrar 2020; Al Jazeera 2020). Local residents increasingly see the refugees of Cox’s Bazar as both a burden on the local economies and an element of insecurity (International Crisis Group 2019). This relocation took place against the backdrop of increasing tensions between local residents and refugees and conflicts between different refugee groups (Hossain 2020; International Crisis Group 2019).

The situation discussed above set the context in which refugee aid agencies need to operate. These challenges warrant a conflict-sensitive humanitarian response in an effort not just to identify sources of tension but also to improve the impact of humanitarian assistance in order to ensure peaceful co-existence between refugee and host communities.

## Evolution of Do No Harm principle

One of the fundamental principles of delivering humanitarian assistance is not to do harm and to guard against unintentionally exasperating current and potential conflicts. Since the 1990s, Do No Harm has gradually emerged as a standard practice for humanitarian actors to avoid inadvertently fueling conflict in delivering aid in a wide range of humanitarian contexts. In 1992, Trócaire, an Irish humanitarian aid agency, spearheaded a rehabilitation program in the war-torn Gedo area of South-Central Somalia and assisted without precipitating the conflict environment (Anderson 1999). Thus, Trócaire became one of the pioneering aid agencies that operationalised the Do No Harm principle’s fundamental tenets, even when Trócaire employees had no training on the Do No Harm principle-based aid operation. Further reference to the Do No Harm principle came in 1993 when the USA and the U.N. decided to withdraw peacekeeping troops from Somalia (Curtiss 1993).

The lessons from Rwandan experience, however, were instrumental in developing the Do No Harm principle (Haider 2014). Lessons learnt from the Rwandan genocide made it evident that a lack of conflict-sensitive international humanitarian aid in Rwanda and Rwandan Refugee camps in Zaire^2^ allowed genocidaires to take advantage of the humanitarian supplies to reinforce their dominance and carry out genocide in Rwanda (Uvin 1998). Later, in the mid-1990s, led by Mary B. Anderson, the Local Capacities for Peace Project (LCPP) was launched to examine the relationship between humanitarian aid and conflict. The term’s genesis is often credited to Mary Anderson’s seminal work “Do No Harm: How Aid Can Support Peace—Or War”, which outlined the original tenets of the framework (Anderson 1999). Also, in the further development of the principle, lessons learned from humanitarian emergencies in various contexts in Bosnia (Reychler 2006), Burundi (Brachet and Wolpe 2005), Sudan (Riak 2000), Sri Lanka (Harris 2010), and Nepal (CDA 2006) have been crucial.

From the 2000s onwards, the Do No Harm principle started achieving widespread acceptance across the field. The Do No Harm principle is now well known in the humanitarian community, and references are found in a range of humanitarian contexts pertaining to international refugee response (Patel 2019), natural disasters (Harris 2006), U.N. peacekeeping (Aoi et al. 2007), peacebuilding (Putzel 2010), humanitarian intervention (Barbolet et al. 2005), post-conflict rehabilitation (Barakat and Zyck 2009), gender mainstreaming (Grabska 2011), and development aid among others (Di Giovanni 2014). Although the premise has been well recognised in the humanitarian community, conflict sensitivity’s^3^ operationalisation represents a significant challenge given the ever-changing nature of the humanitarian space.

## Do No Harm as an analytical framework

The fundamental goal driving the Do No Harm’s increasing use is to stress the need to understand better the actors and the complexities of conflicts to minimise the harm that humanitarian aid may cause with the aid they provide. The use of Do No Harm in this context arose from a profound understanding that, while humanitarian aid is meant to relieve the misery of conflicts and humanitarian emergencies, it can potentially feed into and intensify the rifts between conflicting groups. The principle requires humanitarian agencies to minimise the harm they may cause on account of the aid they provide.

In the context of the Rohingya crisis, humanitarian aid to refugees can facilitate co-existence between the refugees and host communities. However, providing humanitarian aid with good intentions can potentially have adverse effects. According to Anderson (1999), humanitarian aid can affect conflict negatively in two possible ways: it can feed intergroup tensions and weaken intergroup connections. If humanitarian aid impacts conflict in these ways, “it inadvertently exacerbates conflict” (Anderson 1999, p. 69). On the other hand, humanitarian aid can also help conflicts come to an end by reducing inter-group tensions and supporting shared connections between different groups. Thus, the principle aims to evade inadvertently feeding into inter-group conflicts and find and support the dynamics that connect different groups. Incorporating Anderson’s framework within the Rohingya humanitarian response suggests that aid for refugees can cause conflict if it feeds tension between the refugees and locals by further weakening an already fragile inter-group connection.

On the other hand, as Anderson (1999) argued, a conflict-sensitive humanitarian aid could, “ …save lives, reduces human suffering, and supports the pursuit of greater economic and social security in conflict settings.” (p. 67). Although refugees and the host communities are two different social entities, they often develop interdependence where each side provides the other with goods and services that otherwise would have been unavailable. Therefore, humanitarian aid’s role is vital to reduce the probability of tension escalating into a conflict between refugees and the local population. Given this, a contextual analysis of the dividers and connectors between the refugees and host communities is an essential precondition of conflict-sensitive humanitarian aid, particularly in the crisis’ protracted phase.

Before using Do No Harm as an analytical framework in the context of the Rohingya refugee crisis, it is logical to raise a question: “Do no harm to what or whom?” (McInerney-Lankford et al. 2011, p. 46)**.** In this paper, the author focuses on the unintentional impacts of humanitarian aid on poorer hosts and refugee-hosting areas’ overall capacity to respond to the crisis.

## Inadvertent impacts of refugee aid on refugee hosting area

Drawing upon Anderson’s (1999) framework, this section sheds light on the drivers of tensions and major issues that compromise local capacities for peace in the areas hosting refugees. In the context of the refugee-host dynamics in Cox’s Bazar, the causes of tension are not grounded in historical injustice, instead are primarily due to the general lack of effective intervention seeking to address the challenges experienced by the affected locals since the arrival of refugees. The analysis also includes environmental aspects, since the study argues that excluding the environmental dimension of refugee humanitarian aid is a significant omission and an element that needs to be a part of conflict-sensitive refugee response. Further, this section critically examines how humanitarian aid contributes to delocalisation and its link to local peace capacities.

## Refugee aid’s impact on market and service sector

According to Anderson (1999), refugee aid affects the market, which can fuel conflict or reinforce peace. In her study, Jacobsen (2002a) opined that refugees bring a variety of resources in the shape of humanitarian aid, economic opportunities, and human capital. She defined all the resources that follow a refugee crisis as “refugee resources” (Jacobsen 2002a, p. 578). The positive impact of refugee resources is well recognised in refugee literature (Jacobsen 2002a; Alix-Garcia and Saah 2010; Alix-Garcia et al. 2018). However, the presence of aid workers and the supply of humanitarian aid, in the form of either in-kind aid or cash, can have adverse impacts on local markets. The study identified three significant ways in which arrival of aid is impacting local markets: (1) dollarisation of the local economy and flow of in-kind humanitarian aid disrupted local market equilibrium and reinforced powerful local elites’ control over the local economy; (2) the presence of highly paid aid workers and local and international aid organisations caused the cost of living to increase exponentially; (3) and, by diverting significant human capital and skilled employees, aid organisations eroded public service delivery capacities, which in turn made the cost of services higher.

Although in-kind aid is deliberated for refugees, refugee aid inevitably finds its way into the local market (Ground Truth Solutions 2020). An overwhelming majority of the provided aid to the refugees is in-kind. Refugee families sell part of their food items at a reduced rate than the original market price, which allows some locals around refugee camps to buy leaked food items at a lower cost from the informal refugee markets. While such leakage of in-kind aid benefits poorer hosts, host sellers around refugee camps reported decreased profit.

Furthermore, the study identified a clandestine clientele system profiting from refugees selling part of their in-kind aid. Some politically backed local middlemen buy part of the in-kind aid directly from the refugees at a lower price. Collected relief goods are repacked and resold to different aid agencies. Consequently, refugee aid is involuntarily helping to strengthen powerful local elites’ control over resources and those within the supply chain. Such a nexus deprives poorer hosts of refugee aid leakage benefits and further harms the vulnerable local population.

The flow of refugee aid and humanitarian workers has widened the gaps between the “haves and have nots” in Cox’s Bazar. Humanitarian agencies supporting refugees can attract skilled and semi-skilled workers due to attractive salaries (Landau 2004). Rohingya influx provided some locals with new job opportunities with local and international aid agencies (Alsaafin 2018). However, a noticeable presence of aid workers with significantly higher purchasing power caused dollarisation of Cox’s Bazar’s economy with significant adverse impact on the locals. The presence of highly paid local and international humanitarian workers caused the rise of demand for assets and services. For example, cost of housing in Cox’s Bazar district went up by up to 400% in addition to the soaring price of other commodities and services, prompting the government of Bangladesh to declare Cox’s Bazar an expensive town (The Business Standard 2020)**.**

Furthermore, the increase in the cost of almost all essential commodities has placed the residents in Ukhiya and Teknaf in serious financial challenges (The Daily Star 2017). Consequently, local urban middle- and lower-middle-income groups are finding themselves under increasing challenges. Additionally, newly created employment opportunities are mostly available for highly educated development workers and urban elites with accesses to resources. As a result, people who have control over resources and the necessary skills can take advantage of the situation and gain financially. Anderson (1999) argued, “the influx of expatriates bids up the costs of hotel rooms, office space, housing, food, furniture, and equipment. People who own or control these facilities and goods can become wealthy despite deteriorating economic conditions” (p. 43).

In Cox’s Bazar, refugee aid agencies’ hiring practices resulted in skilled human resource shortages, especially in the public service sector (USAID 2018; Anas 2020). Many employees, primarily from the government health and educational sectors, left to take well-paid jobs with different humanitarian agencies, leading to severe public services disruption. While the diversion of public employees provides much needed local knowledge and skills to the humanitarian agencies, the practice harms the government agencies’ service delivery and capacity development. For instance, local community members reported a significant deterioration in healthcare service quality and extended time for healthcare-related service delivery. Consequently, locals are compelled to avail private health services at a significantly higher cost. The situation has become a sore point, as refugees can avail free of cost health care services, whereas locals have to pay a high price for the same services (UNDP 2018). Another adversely impacted service sector is educational activities. In some adversely affected areas, up to 70% teachers have left schools for higher salaried jobs with aid agencies, leading to an alarming level of non-attendance in educational institutions, particularly in Teknaf and Ukhiya (COAST 2018). While these new job opportunities bring short-term financial benefits for some skilled locals, the recruitment practice has changed the local job market’s nature and adversely impacts local public service-oriented institutions’ overall service delivery capacity. Consequently, the cost of essential services such as health and education has increased significantly, making it unaffordable for the poorer hosts. The study has not found any evidence that aid agencies are actively tracking these kinds of adverse market impacts that their operation is having in the refugee-hosting areas.

## Who gets what, when, and how? Distributional impacts of refugee aid

There are decisions regarding who should get aid and who should not have consequences (Anderson 1999). The distributional impact of aid is one of the most critical parts of any humanitarian aid, primarily due to prioritisation and errors in exclusion and inclusion (Devereux et al. 2017). When aid targets some groups at the expense of other vulnerable groups, competition between them results (Anderson 1999). The article identified two broad dimensions of distributional impacts. The first dimension of distributional impact is between the refugees and the locals where poor locals feel that they have been forgotten (ACAPS - Assessment Capacities Project 2018). The second is an intra-host dimension of distributional impact in which socially visible locals get aid, while geographically distant and socially invisible hosts become marginalised or feel overlooked by the humanitarian agencies.

Humanitarian aid distributed among refugees is causing resentment from the part of severely affected host communities. Poorer hosts believe that refugees get most of the aid and plight of the locals do not receive attention, even though they are bearing the brunt of the influx (personal communication, 12 March 2020). Hosts, predominantly in Teknaf and Ukhiya, feel overlooked, although they live under continuous risk due to competition for the same labour market, the growing cost of living, and competition for natural resources. The arrival of the Rohingyas to Cox’s Bazar has raised the level of local poverty by around 52% (The World Bank 2019, p. 386). Many locals in Teknaf and Ukhiya lived in impoverished conditions even before the influx. Poorer hosts often view that refugees have better food safety than many of the disadvantaged hosts. Such an uneven distribution of refugee resources is an obstacle in the path to peaceful co-existence between the impoverished hosts and the refugees. The poorer hosts see legitimate relief aid distributed to refugees as an unmistakable sign of being overlooked. As Anderson (1999) wrote, “differential benefits from aid can reinforce intergroup tensions in conflict areas. When aid targets some groups at the expense of other vulnerable groups, competition between them results,” (p. 46). In addition to this, services created for refugees are not available to the locals. Furthermore, facilities are concentrated near refugee camps, rendering most of them unattainable for the locals.

The second dimension of the distributional problem is fueling intra-host tension, mostly linked to beneficiary selection. The conflicting understanding of beneficiary selection criteria is causing intra-host tension. As Anderson (2000) argued, “If beneficiary selection can worsen intergroup relations, it can also improve them” (p. 25). Hosts living around refugee camps came under aid distribution, but the poorer hosts living far from the refugee camps are the forgotten losers of the refugee influx, as they live in remote places far from the refugee camps. According to a UNHCR-commissioned unpublished baseline survey report, 75.54% of households surveyed in remote villages have never received any support from non-governmental organisations (NGOs) (Center for Natural Resource Studies-CNRS 2019). Aid organisations determine standards to identify who should come under the aid program. However, the beneficiary selection often does not reflect host communities’ ideas of social and economic gaps. The complexity associated with selection makes it relevant to focus on the conceptual aspect of the host community. UNHCR defines a host community as “the country of asylum and the local, regional and national governmental, social and economic structures within which refugees live” (UNHCR 2011). As a group, the host community is often considered as a homogeneous group. This broad definition of the host community does not reflect the full range of challenges that different host community segments face. As Chambers (1986) argued, “Hosts have tended to be a residual, thought of as a single entity summarised as 'host communities', 'the local people' or 'the surrounding population,” (p. 253). Such a generalised host community definition fails to recognise the host community’s different subgroups’ specific needs, particularly the marginal and severely affected host community members living far from the refugee settlements.

Humanitarian aid programs in Cox’s Bazar district, especially in Teknaf, where at least 144 international and local agencies are responding to emergency needs (Alsaafin, 2018), are found to be involved in overlapping and redundancy in resource distribution. Furthermore, the presence of many local and international aid agencies resulted in severe competition for funds and beneficiaries. Such a competition caused inadequate need assessments, the inclusion of non-eligible beneficiaries, and the omission of the aid programs’ eligible poorer hosts. Community representatives also voiced frustration and anger due to the overlapping of beneficiary selection and aid distribution (Community representatives’ meeting, 13 March 2020). Besides, some local public representative claimed that most aid agencies demonstrated tendencies to work in the villages near refugee camps. As a result, the local population living close to refugee settlement received repeated services from the multiple agencies, whereas adversely affected locals living far from the camps and road networks have not received necessary supports. Many agencies are bringing funds from donors, and they want to spend fund quickly and engage with the readily accessible beneficiaries without proper coordination, needs, and sustainability assessments (UNHCR official, personal communication, 12 March 2020). The pressure to show quick results and the impulse to secure funding are two common reasons behind this. Locals also expressed their concerns about divergent approaches and practices of different aid agencies.

Beneficiaries from the host communities have a severe lack of information. Beneficiaries from the hosts do not understand the different distributional criteria and why some people are assisted, and others are not. Such a situation is leading to suspicion of corruption and harming beneficiaries’ relationship with humanitarian agencies. Additionally, the absence of clear communication related to an exit strategy negatively impacts the host communities’ beneficiaries. Some beneficiaries complained about aid’s sudden disappearance, which placed them in deep trouble (beneficiary meeting, 18 March 2020).

Furthermore, in a multi-agency emergency response like the Rohingya crisis, lack of information sharing between different agencies has caused adverse distributional impact and inefficient crisis management. The organisational structure significantly controls the information-sharing mechanism, and agencies are reluctant to share information with other entities. It was observed that lack of information sharing between different humanitarian agencies is one of the key reasons behind negative distributional impacts, poor selection of beneficiaries, and overlap in aid distribution. Such overlapping is impacting social cohesion and contributing to increasing social inequality between different groups of host communities.

### Substitute impact: shifting role of local actors and the issue of legitimacy

The Rohingya refugees have been entering Bangladesh since the 1970s to flee persecution in Myanmar (Crisp 2018). However, before the recent crisis, Bangladesh had not faced such an enormous and quickly unfolding displacement of refugees. At the beginning of the crisis, different local actors, such as host communities, faith-based groups, and local NGOs, provided essential support for the refugees with the local government’s support (Bowden 2018). The remarkable leadership role of local and national actors was visible in the crisis’ initial phase (Wake and Bryant 2018). The U.N. and other international agencies responded to the crisis much later. The latest humanitarian response to the Rohingya crisis has been the largest ever (UNHCR 2019). At the local level, the flow of foreign funding resulted in the change of leadership role. While the U.N. agencies and other international humanitarian organisations introduced much needed financial, logistical, and operation capacity, local actors feel that international agencies have not engaged local actors sufficiently and the decision-making and leadership role moved from local actors to the U.N. agencies and other international organisations (Bowden 2018).

Local humanitarian actors who have been working in the affected region for a long time accused international humanitarian actors of disempowering local actors, such as local NGOs, government bodies, local staff, and previously existed organisations (COAST 2019; The Daily Star 2020). Such a situation resulted in a strong response both from the local actors and the government. The Bangladeshi government accused humanitarian agencies of spending, “no more than 25 per cent of total aid for the refugees” (The Daily Star, 2019b). The similar allegation came from the local actors working in Cox’s Bazar. Coastal Association for Social Transformation Trust (COAST), a Cox’s Bazar-based NGO, conducted a study that claimed international humanitarian actors in Cox’s Bazar spend five times more than the program requirements (The Daily Star 2018). The division between local and international humanitarian actors became even more evident when local civil society organisations and NGOs formed an association named NGO Forum, a platform involving local actors in response to Inter-Sector Coordination Group (ISCG) (CCNF 2019). ISCG performs a coordination function between the Bangladesh government and various foreign NGOs (Banik 2018).

The criticisms against international humanitarian actors can be debated. However, it was evident from the fieldwork that organisations like UNHCR, the International Organisation for Migration (IOM), and Northern humanitarian agencies act as the central bodies due to their authority and control over resources. In contrast, local humanitarian actors work as implementing partners to deliver help and protection to the refugees and host communities. Despite having their mandates, local humanitarian actors merely act as line implementing partners for international donor agencies and other U.N. bodies. Further, local civil society and NGO leadership claimed that local agencies have been working in the affected area for decades, but their skilled employees have been taken away by the U.N. and large international humanitarian agencies (Khan 2019).

As discussed above, the substitution effects have caused delegitimisation of the existing local structure, rendering local takeover even more challenging. The legitimisation effect takes place when humanitarian agencies authorise certain groups or organisations to use resources over others. The presence of humanitarian agencies resulted in the weakening of public service and withdrawal of the government as a service provider, causing the relocation of legitimacy and power to the international aid agencies. Protracted refugee crises continue for decades, and thus, humanitarian missions quickly become a permanent feature of the local social and political fabrics. The continuous presence of aid agencies in Cox’s Bazar rendered public service institutions ineffective and incapable of handling the crisis. The failure to deliver service to the host population has aggravated already fragile host-refugee interaction. The process of delocalisation is also evident in the fund allocation of Rohingya humanitarian response. According to a report, “the majority of funding (69 per cent) goes to U.N. agencies, followed by INGOs (20 per cent) and the Red Cross (7 per cent) and national organisations receive only four per cent” (Khan 2019). Such a disproportionate allocation of financial resources and the international aid agencies’ unwillingness to relegate greater say in financial resource allocation contradicts the pledge made at Grand Bargain (G.B.), Charter for Change (C4C), and Principles of Partnership.

### Do No Harm in refugee response: extending to environment?

It is generally acknowledged that influx of displaced people into a new area can put tremendous pressure on common property resources, prompting adverse environmental and social impacts (Black 1994; Jacobsen 1997; Martin 2005). There are substantial pieces of evidence that large-scale refugee settlement results in environmental degradation, such as the reduction of agricultural and forest land and the depletion of water and other natural resources (Percival and Homer-Dixon 1998; Martin 2005). Homer-Dixon (1994) opined that “environmental change is only one of three main sources of the scarcity of renewable resources; the others are population growth and unequal social distribution of resources” (p. 280). According to Homer-Dixon (1999), in developing countries millions of people, “…tend to be much more dependent on environmental goods and services for their economic well-being; they often do not have the financial, material, and human capital resources to buffer themselves from the effects of environmental scarcities; and their economic and political institutions tend to be fragile and riven with discord” (p. 04). Martin (2005) viewed that struggle over limited natural resources may cause “hardening of group identities and providing a catalyst for hostility towards out-groups” (p. 332). The refugees living in a large settlement have a considerable connection with the environment of the surroundings. As Jacobsen (1997) opined, refugee camps’ settlement is arguably the most significant issue in shaping refugee settlement’s impact on the local environment. She further viewed that, in the absence of alternative income-generating activities, refugees exploit natural resources unsustainably.

As previously stated, a refugee settlement in Cox’s Bazar resulted in adverse environmental changes, including the reduction of agricultural and forest land and depletion of water and other natural resources. Poorer hosts depending on natural resources are not just adversely impacted due to refugees exploiting natural resources, but also due to different aid agencies’ relief operations and establishment of refugee camps. According to the local Agriculture Extension Department, at least 100 ha of cropland in Teknaf and Ukhiya were affected by the refugee situation between August 2017 and March 2018 (UNDP & U.N. Women 2018). Besides that, refugee settlements and humanitarian organisations used 76 ha of cultivable land to set up warehouses and offices (UNDP & U.N. Women 2018). Around 5000 acres of land have become unusable due to sandy soil rolling down from the mountain slopes, which humanitarian agencies have taken for refugee housing purposes (UNDP 2018). The same report stated that grazing lands have reduced. As a result, the number of cattle farms has decreased considerably, by 10–15%. All of these factors contributed to the economic woes of the local hosts dependent on the forests. The scarcity of fresh water for agricultural production has always been a significant concern for the farmers in the affected region. They are mostly reliant on surface water springs, such as hilly streams for irrigation. A report from Energy and Environment Technical Working Group (EETWG) of ISCG found that contamination now exists in more than fourth-fifths of these water sources, owing to Rohingyas’ sudden unplanned settlement (Energy and Environment Technical Working Group 2018).

Humanitarian agencies responding to an emergency refugee crisis harm the refugee hosting areas by excluding environmental consideration at the initial phase of the crisis, as we have seen in the context of Rohingya refugee response. The most severe damage to the environment happened in Cox’s Bazar in the initial phase of the influx when UNHCR constructed emergency relief camps. UNHCR constructed these refugee camps as a transit point or as temporary refugee camps. However, experience from the previous influx and other countries show that temporary refugee camps often become a permanent settlement (Oka 2011; Lui 2007)**.** Several features can be identified from the response of humanitarian agencies to the environment. First, humanitarian aid agencies are less interested in environment management (Kibreab 1999). Environment and natural resource management take long-term planning and financing. The tendency to achieve results quickly sidelines the need for focusing on the environment (Whitaker 2002). Second, environmental policy component is absenting from the part of relief agencies. This can be defined as institutional disinterest to broaden mandate to include environmental concerns. Third, there is no agreement about who should be the custodian of environment and resource management, since UNHCR and other emergency response agencies do not integrate the environment into their emergency aid mechanism. Lastly, identifying refugees as a temporal group with the persistent need for emergency relief prevents refugee aid organisations from cultivating a long-term term plan integrating the environment and surrounding host community (Jacobsen 1997).

## Analysis: ways to support local capacities for peace and challenges to it

Given the findings presented above, it is crucial for refugee aid agencies to support local capacities for peace. There are areas in any conflict environment that connect conflicting groups. While there remains a wide range of challenges, there are two broad policy- and mandate-related constraints that are putting a hold to the conflict-sensitive humanitarian response in Cox’s Bazar. The following sections illustrate factors that can support local capacities for peace and policy- and mandate-related challenges to a Do No Harm principal-based conflict-sensitive response.

### Supporting local capacities for peace and connecting refugees and hosts

The struggle to secure livelihood is central to the ongoing tension between the refugees and the poorer hosts. It is also crucial to recognise that humanitarian aid’s success largely depends on how the local people perceive the possible benefits from the refugee aid offered. Therefore, humanitarian agencies need to widen their livelihood support program and promote self-reliance of the vulnerable hosts. For example, cash for work projects can provide short and mid-term earning opportunities. The alternative livelihood support program is especially needed for the people dependent on natural resources, subsistence farmers, daily wage earners, and fishing communities. Livelihood support programs need to be based on a proper beneficiary selection and need assessment to avoid distributional problems.

The Rohingya humanitarian response can achieve more if it does not see refugees’ necessities as separate from those of the vulnerable hosts. In other words, programs and developments need to be designed to lessen tensions and foster positive relations between the refugees and locals. When applied in the context of a protracted refugee crisis, a Do No Harm-based approach needs to ensure that services and initiatives meet the needs of both the impacted host population and the refugees. One way of mitigating tension and supporting connectors is creating shared service platforms, both for the refugees and hosts living around refugee camps. Humanitarian agencies can create public service platforms for both the refugees and host communities. For example, health care facilities designed for refugees can accommodate hosts living around the refugee camps. Both refugees and hosts can be provided free services from such facilities. Studies from Tanzania show that inclusive development activities in the refugee-hosting areas can help mitigate conflicts between hosts and refugees (Berry 2008).

Despite the Bangladeshi government’s repatriation-centric policy, providing income-generating opportunities for refugees should be a high priority. This will reduce the livelihood conflict between the refugees and poorer host communities (Jacobsen 2002b). Cash for work program for refugees will connect refugees with the local market, potentially benefitting host traders. The market is one of the connectors between the refugees and the host communities. If refugees are given a chance to participate in the local market, both refugees and host can benefit. As the refugees’ movement is regulated, interventions need to be focusing on community-based works within refugee camps. Such programs will improve their well-being, as well as reduce competition in the labour market.

The environmental dimensions of the influx and subsequent humanitarian response need to be seen as an integral part of short-, medium-, and long-term response. While extreme poverty of hosts and refugees is often considered as reasons for a short term, unsustainable exploitation of natural resources, the dependency of poorer hosts and refugees on natural resources can be grounds for preservation and cooperation between different groups. To this end, a Do No Harm-based approach needs to see refugees as productive and innovative contributors, not a burden or exceptional resource degrader. Resource substitution, for example, providing alternative fuel, reforestation of indigenous plants, conservation of sources of water, and giving alternative opportunities for income-generating activities, needs to be the part of a humanitarian action plan. A community-based rainwater harvesting system can be implemented to reduce water conflict between refugees and the locals. Furthermore, the proper distribution of resources, transferring know-how, and improving markets can help locals and refugees to reduce their dependence on natural resources. Additionally, humanitarian agencies need to adopt a participatory environment and resource management approach. Martin’s (2005) study found that in Ethiopia’s Bonga refugee camp, participatory environmental management improved natural resource usage effectiveness while also offsetting tensions between refugees and hosts. A participatory environment and resource management program’s strength is that it does not take environmental and natural resource management in isolation of the beneficiaries. Therefore, such a plan will require long-term environmental planning mainstreamed within refugee aid programs. In addition to an integrated approach to environment and resource management, humanitarian agencies need to work for changing resource use behaviour of the refugees and local population. There has to be a consensus about natural resource utilisation among refugees, host population, and agencies that implement the program.

Supporting local capacity for peace using locally available resources is one of the Do No Harm principle’s central tenets. **S**trengthening local actors’ capacity is essential against the backdrop of compassion fatigue and declining aid for the refugees (Barua 2020). Donor and international humanitarian agencies need to invest both technical and financial resources to build the local actors’ capacity to provide service to the refugees and the host communities. While defining the local and localisation concept is beyond this study’s scope, humanitarian action needs to be, “as local as possible, as international as necessary,” as former U.N. Secretary-General Ban Ki-moon emphasised (United Nations 2016)**.** The localisation process of humanitarian aid can be achieved through direct financing, developing new partnerships, capacity strengthening, coordination, recruitment, and greater communication between local and international humanitarian actors. Therefore, an assessment of international and local agencies’ complementary strengths and weaknesses is necessary to ensure an efficient response. Localising Rohingya humanitarian response should not be seen as a binary opposition to international involvement. Instead, an informed recognition of respective limitations and their complementary strengths will better help identify the needs of the impacted locals and refugees, while also strengthening the local ability to take over the leadership role in humanitarian response incrementally.

Cooperation, collaboration, and information sharing between different agencies are vital to avoid ramifications relating to inaccurate data, overlapping aid, and knowledge inaccuracy. Well-synchronised coordination between humanitarian agencies is expected to improve aid’s outcomes while optimising transparency, productivity, and operational efficiency. Information sharing related to the program’s closing is vital, as premature termination of service can significantly harm beneficiaries. Such an approach will help build trust and, most importantly, allow beneficiaries to plan their options better.

### Do No Harm in Rohingya refugee response: twofold policy challenges?

The outcome of refugee aid depends on a wide range of structural issues and regulations that govern the interactions between refugee aid and beneficiaries in the refugee-hosting areas. Given the Bangladeshi government’s position in the international refugee regime and mandate of the refugee aid agencies, it is crucial to analyse if the dimensions of humanitarian aid agencies’ unintended impacts are partially due to the policy- and mandate-related constraints of the refugee-hosting country and refugee aid agencies.

The first significant impediment to a Do No Harm-based refugee response is that Bangladesh is not a signatory to the 1951 Refugee Convention or its 1967 Protocol (Louis 2019). As a result, the only official policy is the refugees’ repatriation, even though many refugees who fled in 1978 and 1991 are still living in Bangladesh (Mallick 2020). The official policy of the Bangladeshi government is to restrict refugees within camps, leaving very little chance for humanitarian agencies to go beyond emergency refugee relief. Such an approach from the host country is a serious constrain to realising refugee self-reliance. Furthermore, repatriation and relief-centric policy do not allow interventions to foster long-term interactions between the refugees and host communities and bars refugees’ utilisation as productive actors. According to International Crisis Group report (2019), the Bangladeshi government’s “...restrictions on aid programs and its year-to-year approach to planning do not encourage the mobilisation of aid funding for anything beyond the most basic needs of the refugees, let alone host community development” (p. 11).

The Bangladeshi government considers the Rohingya refugee crisis as temporary (Human Rights Watch 2018). This approach has implications for resource management in the refugee-hosting area. Warehousing refugees create exclusionary-driven hazards, such as resentment, distrust, and, in turn, unsustainable usages of the common property resources. Furthermore, such an approach constrains options available for humanitarian agencies in the management of natural resources. The encampment of refugees leads to adverse impacts, such as relief dependence, allocation of scarce land and other natural resources, and unsustainable resource distribution. As Jacobsen (2002b) argued, “viewing refugees as passive victims, who wait for relief handouts and bring only trouble to host countries, fails to see the multiple ways they pursue livelihoods for themselves, and in so doing can contribute to the economic vitality of host areas” (p. 96).

The second stumbling block is related to refugee aid organisations’ mandate, which is institutional disinterest in broadening the mandate to include severely-affected host communities. This institutional disinterest does harm to the poorer host communities. With all its consequences on poorer hosts, the protracted refugee crisis has become a new normal, and the average duration of refugee displacement is estimated to be around 26 years (U.S. Department of State 2016). Therefore, achieving a sustainable solution and peaceful co-existence would require the inclusion of poorer hosts in aid program. Currently, both poorer hosts and refugees face socio-economic challenges because of legal- and mandate-related differences reduced to a binary opposition, with the Bangladeshi government pursuing an unrealistic near-term repatriation policy and aid agencies narrowly focusing on refugees.

Provided the scenario, international humanitarian communities must engage with the government of Bangladesh in constructive dialogue so that Bangladesh can develop and implement comprehensive refugee policy, preferably in line with the International Refugee Convention of 1951. The international community needs to show the Bangladeshi government how encouraging sustainable refugee livelihood can counterbalance many of the refugee-hosting populations’ economic pressures. Such a policy shift would allow refugees to use resources for refugee self-reliance while linking refugees and hosts to the local economic system instead of keeping refugees confined in the camps.

Furthermore, it is equally vital for the humanitarian agencies to recognise that any realistic and sustainable solution will remain elusive without allocating significant if not equal attention to the poorer hosts. UNHCR recognised the need for refugee aid reorientation long ago. A 1983 UNHCR report stated that “international aid needs to be redirected from care and maintenance toward multilateral and bilateral support that encourage refugee self-sufficiency and also address the need of the local population who’s standard of living may be eroded because of the presence of refugee” (UNHCR 1983, p. 15). Thus, humanitarian aid needs to widen the door to include hosts, “...especially those who are poorer, more vulnerable, less visible, less articulate and more likely to be hurt by refugee competition and aid to refugees” (Chambers 1986, p. 260) as part of their conflict-sensitive Do No Harm-based refugee response.

## Conclusion

The delivery of refugee aid in a protracted and complex refugee crisis needs to be grounded on respect for certain core principles. The analysis above reveals that the Do No Harm as a principle and framework of analysis provides a useful tool to identify unintended consequences and intervention gaps in refugee response. There is a clear consensus within the international humanitarian community that harm should be avoided in humanitarian undertakings. However, the Do No Harm principle so far remains a precautionary, non-binding principle that is inadequately addressed in passing in humanitarian aid literature (Giovanni 2014). A closer look at the humanitarian aid literature reveals that the international humanitarian community has not developed systematic accountability and responsibility-sharing mechanism to offset humanitarian aid driven unintended impacts. As Giovanni (2014) argued, “. . . a clear indication of the consequences for causing harm - who should be accountable to whom and how - is largely absent” (p. 205).

The author identified certain crucial aspects that need to be incorporated within the refugee humanitarian response to translate the Do No Harm principle into a useful hands-on tool for doing good in refugee humanitarian undertakings. While the application of the Do No Harm principle is highly context-specific, the key components emerging from the analysis above include the following: (a) humanitarian agencies need to understand the context of the refugee-hosting area and how their interventions impact both refugees and host communities; (b) while Rohingya refugees deserve full-fledged support, a Do No Harm-based humanitarian aid needs to identify disadvantaged and excluded poor hosts. Refugee humanitarian aid that includes both refugees and poor hosts are more likely to yield good results; (c) humanitarian aid needs to support capacity building of the local actors instead of developing a parallel system that undermines the legitimacy of the local authority and impairs service delivery capacity; (d) a Do No Harm-based approach requires humanitarian agencies to provide beneficiaries information related to goods and services. For example, aid closure- and exit strategy-related information should be communicated; (e) well-defined accountability and responsibility-sharing framework must be an essential component of a Do No Harm principle-based humanitarian aid; (f) an independent body consisting of appropriate local and international stakeholders should track and determine the efficacy of refugee humanitarian aid. The body should be in a position to identify intervention gaps and place recommendation based on evaluation; (g) environmental impact of humanitarian response needs to be anchored within the operational framework of the aid agencies; (h) in order to avoid the implications of redundancy and information inaccuracy, collaboration, coordination, and information exchange between different humanitarian agencies are essential.

In the absence of the components highlighted above, the Do No Harm principle will only remain a non-binding, normative principle that cannot exercise any meaningful impact to alleviate harm inflicted on people who are already vulnerable. Therefore, the article argues that humanitarian agencies should not just limit themselves to identifying the unintended consequences and lapses in the intervention. Instead, the Do No Harm principle should lead refugee aid agencies to make an active effort to accept responsibility for the harm while taking all necessary steps to mitigate or avoid harming in future interventions.

To conclude, an analysis of the unintended consequences of refugee aid on the refugee-hosting area is not an attempt to suggest circumvention of humanitarian aid. As Anderson (1999) wrote, “it is a moral and logical fallacy to conclude that because aid can do harm, a decision not to give aid would do no harm” (p. 02). Evaluating how refugee aid may inadvertently harm poorer hosts should help refugee aid agencies to be more responsive to potential unintentional consequences and program humanitarian response in a way that Do No Harm to poorer hosts and the long-term prospect of refugee-hosting areas’ capacity to self-sustain. Furthermore, understanding the nature of the direct and indirect impact of refugee aid, despite the positive intentions, will enable humanitarian agencies to formulate suitable policy response. To this end, it is essential to identify potential sources of conflict, assess local capacity for peace, and finally set aid priorities imbued in a conflict-sensitive humanitarian approach to mitigate brewing tension between the refugees and the host community. Therefore, this study attempts to remind aid agencies of the importance of ensuring an environment where refugees do not become scapegoats for the poorer host communities’ challenges.

## Data Availability

On request from the corresponding author, Abu Faisal Md. Khaled the data supporting the findings of this study are available. Due to their containing information which may violate the confidentiality of study participants, the data is not publicly accessible.

## Footnotes

1 Cox’s Bazar district borders the Rakhine State of Myanmar on the southeast coast of Bangladesh.

2 Currently known as the Democratic Republic of the Congo

3 “Do No Harm” is the first principle ideally illustrated in Conflict Sensitivity literature
